# Supplementary material for: Thyroid Transcriptomic Profiling Reveals the Follicular Phase Differential Regulation of lncRNA and mRNA Related to Prolificacy in Small Tail Han Sheep with Two FecB Genotypes
Source: Genes (Basel). 2022 May 10;13(5):849. doi: 10.3390/genes13050849 (PMC9141851; doi:10.3390/genes13050849)
Supplement: Supplementary file 1 [file genes-13-00849-s001.zip › Description of supplementary materials.pdf]

Supplementary Table S1: Total set of DELs was up-and-down-regulated in two groups.

Supplementary Table S2: Total set of DEGs was up-and-down-regulated in two groups.

Supplementary Table S3: Total set of DELs_targets were up-and-down-regulated in two groups.

Supplementary Table S4: GO enrichment of differentially expressed DELs_targets in two groups.

Supplementary Table S5: GO enrichment of differentially expressed DEGs in two groups.

Supplementary Table S6: KEGG enrichment pathways for differentially expressed DELs_targets in two groups.

Supplementary Table S7: KEGG enrichment pathways for differentially expressed DEGs in two groups.

Supplementary Table S8: Co-expression details of DE lncRNA-mRNA after DELs_targets coincided with DEGs in two groups.

Supplementary Table S9: QPCR data of DELs and DEGs in two groups
